# Supplementary material for: Phlebotomus papatasi Antimicrobial Peptides in Larvae and Females and a Gut-Specific Defensin Upregulated by Leishmania major Infection
Source: Microorganisms. 2021 Nov 6;9(11):2307. doi: 10.3390/microorganisms9112307 (PMC8625375; doi:10.3390/microorganisms9112307)
Supplement: Supplementary file 1 [file microorganisms-09-02307-s001.zip › Figure_S1 - Kykalova et al.pdf]

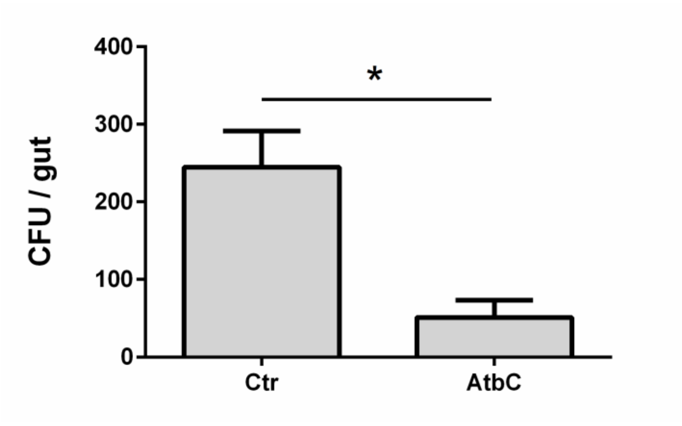

Figure S1: Effects of AtbC-treatment on CFU from sugar-fed *P. papatasi* dissected guts. CFU counted from homogenized dissected guts in sterile saline solution, plated on blood agar, and incubated for 48 h at 25 °C. The y-axis indicates the number of CFU per sand fly gut. The x-axis indicates non-treated sand fly control group (Ctr) and AtbC-treated groups. The significant difference was calculated using the t-test analysis (* $p < 0.05$).
