# Supplementary material for: Phlebotomus papatasi Antimicrobial Peptides in Larvae and Females and a Gut-Specific Defensin Upregulated by Leishmania major Infection
Source: Microorganisms. 2021 Nov 6;9(11):2307. doi: 10.3390/microorganisms9112307 (PMC8625375; doi:10.3390/microorganisms9112307)
Supplement: Supplementary file 1 [file microorganisms-09-02307-s001.zip › Figure_S2 - Kykalova et al.pdf]

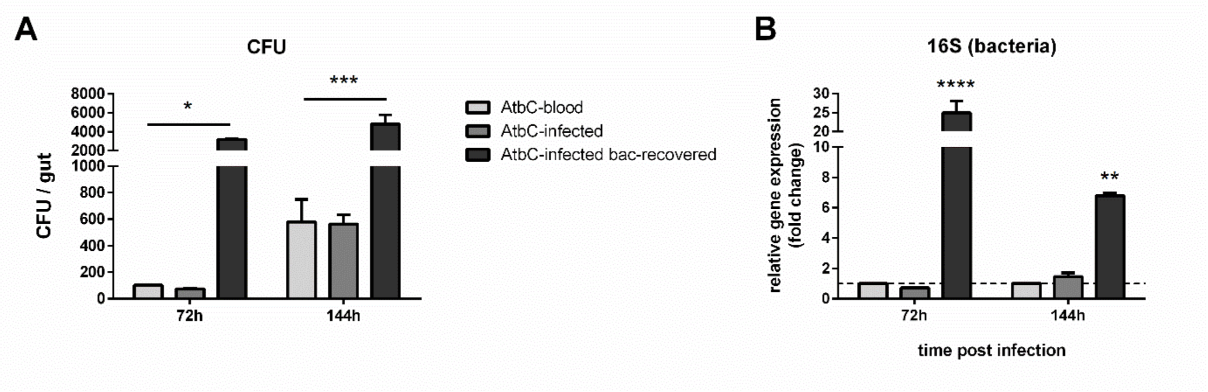

Figure S2: Effects of AtbC-treatment based on CFU calculation and qPCR bacteria detection from treated and non-treated females. (**A**) CFU counted from blood agar plates. The y-axis indicates the average of CFU per sand fly gut counted from triplicate plating. (**B**) Bacteria 16S rRNA expression by qPCR. The y-axis represents the relative gene expression from dissected guts plotted as fold change values compared to the non-infected control group (AtbC treated). The x-axis indicates the collection time-points. Light grey bars indicate the control group fed on blood containing AtbC (non-infected and bacteria-reduced), mid grey bars indicate *Leishmania* infected flies treated with AtbC (infected and bacteria-reduced), and dark grey bars indicate *Leishmania* infected flies without AtbC (infected and bacteria-recovered). Vertical bars represent the average values of three independent experiments, and error bars represent the standard error. Two-way ANOVA test was carried for significant differences (* $p < 0.05$; ** $p < 0.01$; *** $p < 0.001$; **** $p < 0.0001$).
