# Supplementary material for: Phlebotomus papatasi Antimicrobial Peptides in Larvae and Females and a Gut-Specific Defensin Upregulated by Leishmania major Infection
Source: Microorganisms. 2021 Nov 6;9(11):2307. doi: 10.3390/microorganisms9112307 (PMC8625375; doi:10.3390/microorganisms9112307)
Supplement: Supplementary file 1 [file microorganisms-09-02307-s001.zip › Figure_S3 - Kykalova et al.pdf]

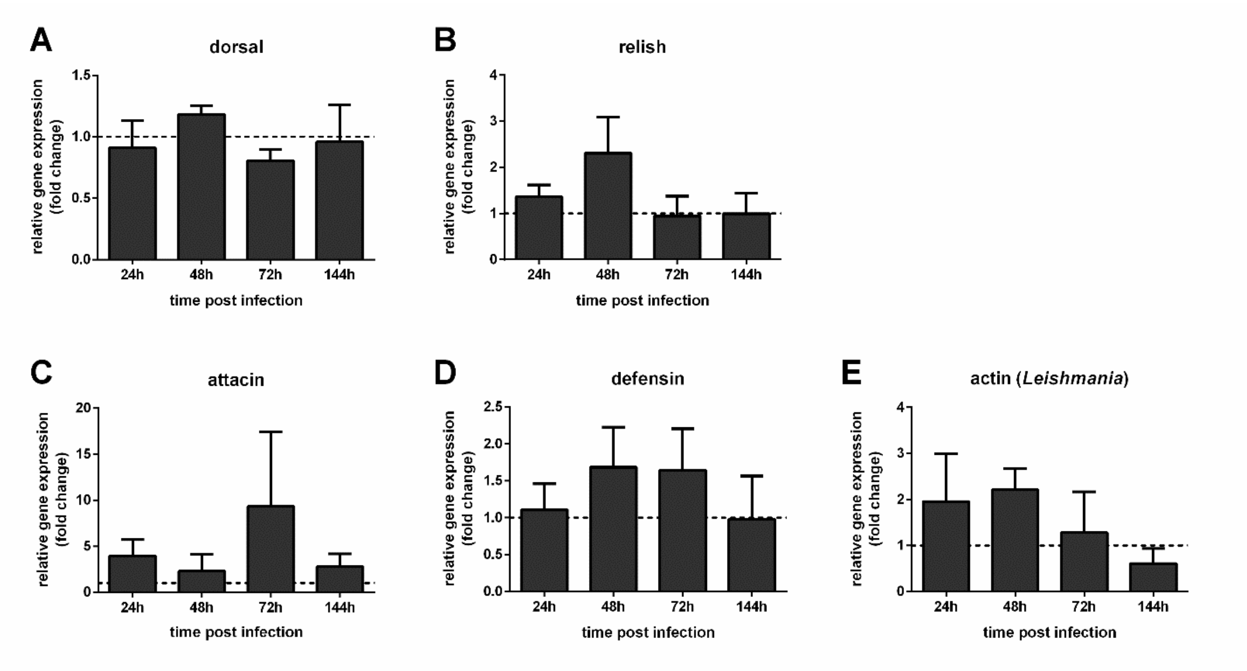

Figure S3: **Relative gene expression of immunity genes in *Leishmania*-infected females with recovered gut bacteria compared to infected females treated with AtbC**. (**A**) Dorsal; (**B**) relish; (**C**) attacin; (**D**) defensin; (**E**) *Leishmania* actin. The y-axis represents relative gene expression as fold change values of *Leishmania*-infected females with recovered gut microbiota compared to infected females treated with AtbC (dotted line) collected at the corresponding time points. The x-axis represents females collected at different time points post-infection. Vertical bars represent the average values of three independent experiments, and error bars represent the standard error. No significant differences were found (two-way ANOVA).
