# Supplementary material for: Phlebotomus papatasi Antimicrobial Peptides in Larvae and Females and a Gut-Specific Defensin Upregulated by Leishmania major Infection
Source: Microorganisms. 2021 Nov 6;9(11):2307. doi: 10.3390/microorganisms9112307 (PMC8625375; doi:10.3390/microorganisms9112307)
Supplement: Supplementary file 1 [file microorganisms-09-02307-s001.zip › Table_S1 - Kykalova et al.pdf]

**Table S1**: L3 and L4 larvae growth period under different feeding regimen

| | elapsed days between L3 and early-L4 stages | | elapsed days between early L4 and late-L4 stages | |
|---|---|---|---|---|
| larvae food | mean | standard deviation | mean | standard deviation |
| autoclaved | 7.000 | 0.000 | 7.000 | 0.000 |
| microbe-rich | 4.500 | 0.707 | 6.000 | 1.414 |

Data obtained from two independent experiments
